# Effect of the EBM-integrated BOPPPS model on clinical competence and EBM confidence in neurology clerkships for three-year junior college medical clerks

**DOI:** 10.3389/fpubh.2025.1676073

**Published:** 2025-10-13

**Authors:** Yaxi Chen, Zhanqin Xiao, Xiang Gu, Qi Lang, Chanxi Chen, Junhuai Zhang

**Affiliations:** ^1^School of Clinical Medicine, Chongqing Medical and Pharmaceutical College, Chongqing, China; ^2^Integrated Diagnosis and Treatment Center for Neurological Diseases, The People’s Hospital of Chongqing Yubei District, Chongqing, China

**Keywords:** evidence-based medicine, BOPPPS, neurology, clerkship, OSCE

## Abstract

**Background:**

Neurology clerkships are critical for clerks’ transition from student to assistant physician, but complex neurological content and traditional lecture-based teaching often reduce learning enthusiasm and skill mastery.

**Objectives:**

This study aimed to evaluate whether integrating Evidence Based Medicine into the BOPPPS teaching model (EBM-BOPPPS) can enhance the clinical competence and EBM confidence of three-year junior college medical clerks during neurology clerkships, in comparison to the standalone BOPPPS model.

**Methods:**

A mixed-method research approach was adopted, with its core quantitative component being a stratified randomized controlled trial with quasi-experimental design. A total of 97 three-year junior college medical clerks were recruited and randomly assigned to the EBM-BOPPPS group (*n* = 47) or standalone BOPPPS group (*n* = 50). Outcomes were measured via a modified OSCE (4 stations, ICC = 0.87), a 22-item EBM confidence survey tailored for junior college clerks (3-point scale, Cronbach’s *α* = 0.76), and MCQs for foundational neurological knowledge. Statistical analyses included independent samples t-test, Mann–Whitney U test, and Bonferroni correction (corrected *α* = 0.003).

**Results:**

At baseline, the two groups showed no significant differences in gender, age, epidemiology scores, core medical course averages or pre-rotation MCQs scores. Post intervention, MCQs scores remained comparable between groups. However, the EBM-BOPPPS group achieved significantly higher total OSCE scores (91.65 ± 2.54 vs. 88.86 ± 4.19, *p* < 0.001) and Physical Interview station scores (20.82 ± 1.56 vs. 19.64 ± 1.78, *p* = 0.001), with both results retaining significance after Bonferroni correction. For EBM confidence, the EBM-BOPPPS group showed a significant pre-post increase in total scores (baseline: 20.1 ± 2.8 vs. post: 30.2 ± 3.3, *p* < 0.001), particularly in understanding EBM concepts. In satisfaction surveys, the EBM-BOPPPS group showed significantly better outcomes in “develop problem-solving skills” (*p* = 0.003), “formulating clinical questions (*p* = 0.001), “critically appraising journal articles” (*p* = 0.003), and “recognizing EBM’s future career importance” (*p* = 0.001).

**Conclusion:**

The EBM-integrated BOPPPS model effectively enhances the clinical competence and EBM confidence of three-year junior college medical clerks, better aligning with the training needs of grassroots primary care compared to the standalone BOPPPS model. Future studies should focus on long-term skill retention and optimizing the model to reduce perceived learning burden.

## Introduction

The three-year junior college medical program plays a crucial role in medical education in China, as it is responsible for training prospective healthcare professionals to provide care in village health clinics and township hospital (1, 2). The clerkship for three-year junior college medical students in their final clinical clerkship year, typically referred to as “medical clerks” in clinical education settings, marks a significant transition from being a student to becoming an assistant physician. However, the clerkship experiences of these junior medical clerks are rarely examined. Neurology, encompassing neurodegenerative and cerebrovascular diseases, requires an understanding of neuroelectrophysiology, anatomy, and imaging for accurate diagnosis and treatment (3).

Anatomical and physiological complexity, the non-specific nature of symptoms and the inherent diagnostic uncertainty and heavy reliance on evidence-based guideline application make neurology learning challenging for clerks, leading to diminished learning enthusiasm and insufficient mastery of relevant knowledge (4, 5). Given this context, traditional lecture-based, teacher-centered methods are inadequate for teaching about nervous system diseases. Ongoing reforms are necessary to enhance instruction and address the demand for skilled medical practitioners.

According to the *British Columbia Institute of Technology* in Canada, the BOPPPS teaching model is designed to establish a structured instructional sequence comprising the following stages: **B**ridge-in, **O**bjective, **P**re-assessment, **P**articipatory Learning, **P**ost-assessment, and **S**ummary (6). This pedagogical approach has been widely adopted in China due to its effectiveness in stimulating students’ interest in learning and enhancing their autonomous learning capabilities and academic performance (7–10). Nonetheless, while the BOPPPS model excels in optimizing the instructional process, it exhibits limitations in guiding the depth of content delivery and fostering clinical reasoning. For example, during the Participatory Learning phase, if case discussions lack a thorough exploration of why a particular treatment plan is prioritized, students may only comprehend the conclusions without fully understanding the underlying evidence hierarchy and the conditions under which these conclusions are applicable.

Evidence-based medicine (EBM) first took shape in the 1990s, aiming to weave together cutting-edge epidemiological findings and research results with the realities of modern clinical practice. Its core mission has been to translate epidemiological principles and methods into tangible tools for daily patient care (11). EBM follows a five-step process: formulating focused clinical questions, systematically searching for relevant evidence, rigorously evaluating the quality of that evidence, integrating it with individual patient values and preferences, and applying it in practice (12). Introducing EBM to medical clerks can help them discern the reliability of medical information. Moreover, it enables them to break free from the limitations of relying solely on experience or textbooks in future clinical practice, providing more precise and personalized medical services supported by scientific evidence, and ultimately achieving a leap in clinical diagnosis and treatment capabilities (13–18). Recent systematic reviews have confirmed the BOPPPS model’s efficacy in enhancing medical students’ clinical skills (19). However, few studies have integrated EBM into the BOPPPS framework, particularly in three-year junior college medical clerkships, creating a gap our study aims to address.

The Objective Structured Clinical Examination (OSCE) is a hands-on assessment approach where examinees rotate through structured stations, each with specific tasks, while examiners use standardized scoring criteria to evaluate their clinical skills. This method has gained widespread adoption across the globe (20). In this study, we integrated the EBM focused BOPPPS model within the context of neurology clerkship education and assessed the clerks’ clinical competence using a modified OSCE. By incorporating clinical medical knowledge through specialized courses, our objective was to impart the concept of EBM to students, stimulate their enthusiasm for learning, and conduct a preliminary evaluation of the instructional effectiveness.

## Methods

### Design

A mixed-method research approach, incorporating a quasi-experimental study design alongside descriptive qualitative research, was employed. Sample size calculation was performed using G*Power 3.1.9.7 software. A medium effect size (Cohen’s *d* = 0.5) was assumed for the primary outcome. With a significance level (*α*) set at 0.05 and power (1 − *β*) at 80%, the calculated minimum sample size per group was 44. After accounting for a 10% anticipated dropout rate, the study involved 97 medical clerks from a three-year junior college medical program at Chongqing Medical and Pharmaceutical College, selected as participants from July 2024 to May 2025. These students participated in a 2-month clerkship rotation in the Department of Neurology, which included a scheduled 50-min class every Wednesday or Thursday afternoon. Prior to the clerkship, all students had completed a course in Epidemiology.

Participants were allocated using stratified randomization to ensure baseline balance. Stratification factors included gender and pre-rotation epidemiology course scores, which served as a proxy for baseline EBM knowledge. Students were ranked by epidemiology scores within each gender stratum, divided into blocks of 4 (with 2 students randomly assigned to each group within each block), and randomly assigned to the EBM-BOPPPS group (*n* = 47) or BOPPPS group (*n* = 50) using computer-generated random numbers. Baseline characteristics of the two groups are presented in Table 1. Baseline characters included gender, pre-rotation epidemiology course scores and assessment of core medical course average covering anatomy, physiology, and pathophysiology to reflect overall academic readiness. No significant differences were observed in gender (*p* = 0.710), age (*p* = 0.559), epidemiology course score (*p* = 0.763), or core medical course average (*p* = 0.621).

**Table 1:** Comparison of baseline characteristics between EBM-BOPPPS and BOPPPS groups.

Variable	EBM-BOPPPS (*n* = 47)	BOPPPS (*n* = 50)	*p* Value	Effect size
Gender, *n* (%)			0.710	Cramer’s *V* = 0.04
Male	18 (38.3%)	21 (42.0%)		
Female	29 (61.7%)	29 (58.0%)		
Age (years)	20.13 ± 1.16	19.98 ± 1.24	0.559	Cohen’s *d* = 0.12
Epidemiology course score	80.84 ± 6.56	80.84 ± 6.41	0.763	Cohen’s *d* = 0.00
Core medical course average	78.62 ± 5.31	79.15 ± 4.89	0.621	Cohen’s *d* = 0.10

Both groups completed epidemiology courses and had basic EBM exposure (per the three-year junior college curriculum).

Effect size interpretations: Continuous variables: Cohen’s *d* < 0.2 (small); Categorical variables: Cramer’s *V* < 0.1 (small). All *p* > 0.05 indicate no significant between-group differences.

Notably, per the three-year junior college medical program curriculum, all participants had completed a pre-rotation Epidemiology course that included a 1-h literature search module, which covered basic digital literacy skills critical for EBM practice, such as navigating the internal medical database, selecting keywords for evidence retrieval, and distinguishing between basic literature types. While formal module scores or standardized digital literacy assessments were not collected as baseline data, this foundational training ensured all participants had minimal digital literacy proficiency, reducing the potential for large baseline disparities in database-related abilities between the two groups. Additionally, both groups had received basic EBM exposure through the same curriculum, eliminating baseline bias arising from prior EBM-related knowledge gaps.

The primary outcome was predefined as the total score of the modified OSCE, as it directly reflects the integration of clinical skills and EBM application, which are core competencies targeted by the EBM-BOPPPS model. Secondary outcomes included scores of individual OSCE stations, MCQs scores for knowledge acquisition, satisfaction questionnaire results for learning attitude, and confidence in EBM concepts from pre- and post-rotation surveys.

### Intervention

#### Experimental group

**Figure 1:**
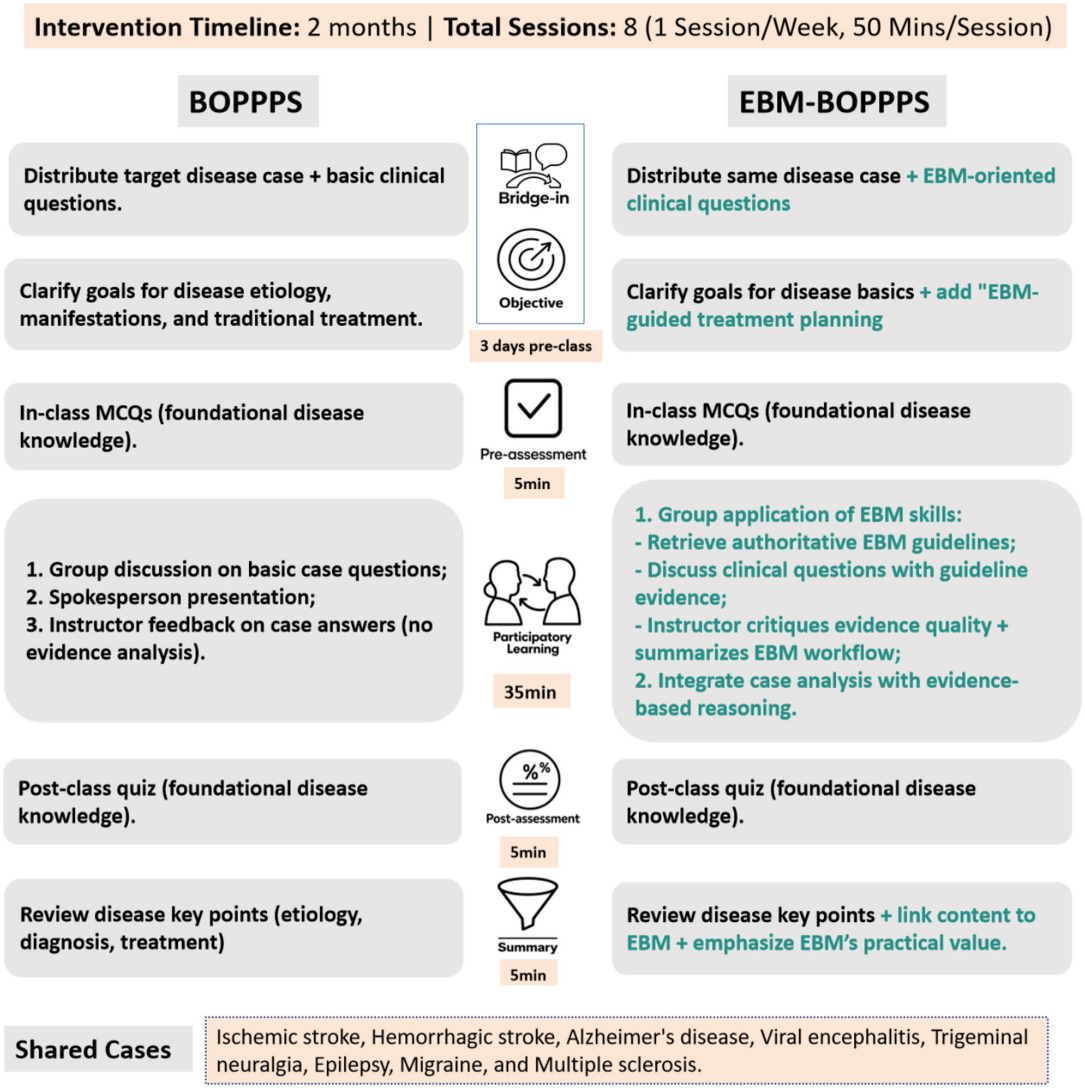
Standardized process comparison of EBM-BOPPPS vs. Standalone BOPPPS models. This figure illustrates the step-by-step standardized workflow of two educational Intervention models implemented in a 2-month neurology clinical clerkship (July 2024 to May 2025). Both models followed a 50 min session format, With I session per week (8 total sessions) and 8 neurological cases integrated into participatory learning (Ischemic Stroke, hemorrhagic stroke, Alzheimer’s disease, viral encephalitis, trigeminal neuralgia, epilepsy, multiple sclerosis, migraine). EBM, Evidence-Based Medicine; BOPPPS, Bridge-in, Objective, Pre-assessment, Participatory Learning, Post-assessment, Summary.

EBM-BOPPPS group was designed by incorporating EBM principles with BOPPPS procedure to form a hybrid teaching method. The instructor employed online learning platforms to provide evidence-based instruction to students on the fundamental skills of medical literature search and evaluation. In the initial week of the neurology clerkship, a two-hour offline session was dedicated to EBM training, wherein students acquired knowledge on literature search techniques and EBM-related principles. This training aimed to equip students with the ability to procure the most valuable references, treatment guidelines, and pertinent research advancements for the purpose of diagnosing and treating diseases. The model was applied through the following six stages (Figure 1). *Ischemic Stroke* was used as an example to display this teaching model (Supplementary Table 1). Key stages included:

1. **Bridge-in (B):** Three days pre-class, students received a case of a 65-year-old male with abrupt right limb weakness, dysarthria, and right Babinski sign, paired with 5 EBM-focused clinical questions to trigger pre-class analysis.
2. **Objectives (O):** Aligned with the case, learning objectives focused on mastering ischemic stroke’s etiology, clinical manifestations, and EBM-guided therapeutic principles.
3. **Pre-assessment (P):** The instructor assessed the students’ knowledge comprehension through the employment of several multiple-choice questions at the beginning of class.
4. **Participatory learning (P):** Groups conducted evidence-based analysis: (a) Using pre-trained retrieval skills to access the Ischemic Stroke Guidelines; (b) Discussing answers to the 5 clinical questions; (c) Appointing representatives to present treatment strategies. The instructor then synthesized group findings, critiquing evidence quality and summarizing the EBM workflow.
5. **Post-assessment (P):** The instructor assessed the students’ knowledge comprehension through the completion of online quizzes at the conclusion of the class.
6. **Summary (S):** The instructor integrated case insights to summarize ischemic stroke’s etiology, diagnosis, and prognosis, emphasizing how EBM principles resolved the 5 clinical questions.

#### Control BOPPPS group

At the outset, the instructor introduced a medical case along with related questions, objectives, and a pre-class quiz, mirroring the approach used in the EBM-BOPPPS group, which includes **B**ridge-in, **O**bjectives, and **P**re-assessment components. The class commenced with a brief topical overview and agenda outline by the instructor. Groups then engaged in a collaborative analysis of the case, aiming to address the posed questions. Each group designated a spokesperson to deliver a presentation of their proposed solutions. Following these presentations, the instructor provided constructive feedback on the groups’ discussions, offered a comprehensive analysis of the case, and addressed lingering queries. The session then transitioned to online quizzes, aligning with the **P**articipatory Learning and **P**ost-assessment components of the instructional framework. The instructor concluded with a synthesized **S**ummary of the disease.

#### Educational intervention

To ensure implementation fidelity, all four instructors completed a 2-h training workshop covering standardized delivery of intervention components, with certification via a sample session assessment. A detailed teaching manual provided scripted guidance, timelines, and case materials for all stages (Supplementary teaching manual). Biweekly peer review meetings and random audio-recording of 20% of sessions were scored via a fidelity checklist and confirmed consistent delivery, with an average adherence of >4.5/5. The instructors delivered both the EBM-BOPPPS and BOPPPS interventions, following strict separation of lesson plans and materials for each group.

To assess the additional time burden of EBM integration, the four instructors first established a baseline preparation time for the standalone BOPPPS model during the 2 weeks prior to the intervention and this baseline (excluding in-class teaching hours) included drafting case outlines, designing pre/post-assessments, and organizing group discussion materials, with a mean of 3.5 ± 0.5 h/week. During the 8-week intervention, instructors maintained weekly teaching logs to record extra time spent on EBM-specific tasks: (1) Curating EBM resources: 0.8 ± 0.3 h/week; (2) Adapting BOPPPS stages to EBM logic: 0.7 ± 0.2 h/week; (3) Preparing EBM training materials: 0.5 ± 0.2 h/week. The total extra time per week ranged from 1.4 to 2.0 h across instructors, with a group average of 1.8 ± 0.4 h/week.

Instructors completed standardized training to avoid differential treatment, with weekly fidelity checks confirming adherence to group-specific protocols. To prevent cross-group contamination, three measures were established to avoid information exchange between groups: distinct class schedules (EBM-BOPPPS sessions conducted on Wednesdays, BOPPPS sessions on Thursdays) with no overlapping time slots; dedicated physical classrooms to minimize informal interactions between groups; pre- and post-intervention agreements signed by students, coupled with verbal reminders before each session, to refrain from discussing intervention content with peers from the other group.

### Outcome measurement

#### Confidence in evidence-based medicine principles knowledge

At both the commencement and conclusion of the rotation, students in the EBM-BOPPPS group were surveyed and evaluated concerning their preferred resources for addressing challenges encountered during the learning process. These resources included medical websites, textbooks, online learning platforms, general search engines, and evidence-based guidelines. Additionally, within the EBM-BOPPPS cohort, a survey was conducted both before and after the rotation to assess students’ confidence in understanding 10 statistical terms: standard deviation, confidence limits, odds ratio, Chi-square test, Student’s t-test, ANOVA, normal distribution, sensitivity, hypothesis testing, and descriptive statistics. Moreover, 12 concepts pertinent to EBM were also evaluated: sample size, MeSH term, stratification, loss to follow-up, prevalence, dropout, study quality, clinical guideline, meta-analysis, randomization, blinding, and PICO element. For each of these 22 items, respondents were provided with three response options: 0 indicating no confidence, 1 indicating moderate confidence, and 2 indicating high confidence. To ensure the scale’s validity and reliability, supplementary analyses were conducted:

(1) **Content validity:** Given the absence of official EBM competency guidelines for Chinese junior college medical clerks, two senior EBM instructors with more than 8 years of medical education and clerkship supervision experience evaluated the relevance of the 22 items against a composite framework (detailed in Supplementary Table 2), which integrated three evidence-based components: (1) Core EBM steps (Ask → Acquire → Appraise → Apply → Assess) and learning domains from Kumaravel et al. (21); (2) Clinical practice needs of grassroots hospitals in China; (3) EBM-related requirements outlined in The Teaching Standards for three-Year College Clinical Medicine Programs (2023). A 4-point relevance scale was used (1 = Not relevant to 4 = Highly relevant), with instructors rating items independently. The final content validity index (CVI) was 0.84 (exceeding the acceptable threshold of 0.80), and all items achieved an item-level CVI ≥ 0.75, confirming no item was irrelevant to the target population.
(2) **Internal consistency:** A pilot test was first conducted with 20 three-year college neurology clerks to assess the scale’s internal consistency. Cronbach’s *α* was 0.78 for the total scale, with subscale α values of 0.75 (statistical terminology) and 0.79 (EBM concepts). For the main study sample (*n* = 47), Cronbach’s *α* remained stable at 0.76.
(3) **Construct alignment:** To verify whether scale scores reflected real-world EBM application skills, a post-hoc analysis was performed to correlate total EBM Confidence Scale scores with performance on the OSCE Physical Interview station which requires EBM-guided history-taking. A moderate positive correlation was observed between the two variables (*r* = 0.35, *p* < 0.01), indicating the EBM Confidence Scale effectively captures a construct relevant to practical EBM competency.

#### Satisfaction with teaching

Both groups were asked to complete an anonymous online questionnaire at the end of the rotation, using a five-point Likert scale (1 = Strongly disagree, 2 = Disagree, 3 = Neutral, 4 = Agree, 5 = Strongly agree) to assess satisfaction with teaching quality (see Supplementary Students Questionnaire for full items). The questionnaire comprised 11 items, covering three core dimensions aligned with study outcomes.

Learning attitude and general competencies: (1) “It is easy to know the learning goals”; (2) “The course helps enhance my learning motivation”; (3) “The course develops my problem-solving skills”; (4) “The course promotes the memorization of knowledge”; (5) “The course improves my communication skills”; (6) “The course improves my ability to give presentations”.

EBM-specific skills: (7) “I can formulate a clinical question to search for evidence”; (8) “I am confident in critically appraising a journal article”; (9) “I consider evidence based medicine important to my future career”.

Learning burden and stress: (10) “I consider this course taking up too much of my preparation time”; (11) “I consider the preparation and presentation for this course is quite stressful for me.”

#### Knowledge acquisition

##### MCQs

At the commencement of the neurology rotation, all students were mandated to undertake a pretest, which was scored out of a maximum of 100 points and comprised 50 multiple-choice questions (MCQs) aimed at evaluating foundational medical knowledge. Upon the conclusion of the rotation, the evaluation of neurology knowledge acquisition was conducted via an assessment consisting of MCQs worth 100 points and an OSCE also valued at 100 points. The MCQs focused on fundamental concepts related to nervous system disorders, whereas the OSCE section encompassed illustrative cases including ischemic stroke, hemorrhagic stroke, Alzheimer’s disease, viral encephalitis, trigeminal neuralgia, epilepsy, migraine, and multiple sclerosis.

##### OSCE

Clinical competence was evaluated using a modified OSCE tailored to neurology clerkships, with four stations and a structured scoring checklist detailed in Supplementary Table 3. Each station was allocated 5–8 min and scored on domain-specific criteria (25 points per station, total 100 points for all stations):

**Station 1** (Physical Interview,): Onset inquiry (5 points), associated symptoms (5 points), past history and risk factors (5 points), logic and efficiency (5 points), and communication attitude (5 points). Dr. Xiao served as the examiner. **Station 2** (Physical Examination): Exam relevance (8 points), technical correctness (8 points), result accuracy (5 points), and patient comfort (4 points). Dr. Lang was the examiner. **Station 3** (Clinical Judgment): Primary diagnosis (5 points), differential diagnosis (5 points), imaging or lab interpretation (8 points), and next-test recommendations (7 points). Dr. Gu conducted the assessment. **Station 4** (Communication Skills): Information accuracy (10 points), clarity (8 points), and empathy (7 points). Dr. Zhang was the examiner.

The OSCE validity and reliability were rigorously established as follows:

**Content validity**: The four stations were reviewed by a panel of three senior neurologists with more than 10 years of clinical and teaching experience and two medical education experts against the core competencies outlined in the Neurology Clerkship Training Guidelines of China Medical Education Association. The panel confirmed comprehensive coverage of key domains, including history collection, neurological examination, evidence-based decision-making, and patient communication, with a content validity index of 0.92.

**Construct validity**: A pilot study (*n* = 20) demonstrated that OSCE total scores were significantly higher in resident doctors who had completed standardized training than in postgraduate students who had no formal clinical clerkship experience in neurology (85.2 ± 4.3 vs. 72.6 ± 5.1, *p* < 0.001), supporting its ability to differentiate between varying levels of clinical competence.

**Reliability**: Scoring was conducted based on a structured checklist (Supplementary Table 3). Four attending physicians (Dr. Xiao, Dr. Lang, Dr. Gu, Dr. Zhang) served as examiners, with measures to minimize bias. Assessors were blinded to group allocation and scored strictly according to checklist criteria without access to group affiliation data. To ensure inter-rater reliability, all examiners completed a 2-h pre-assessment calibration workshop including joint review of the checklist, practice scoring with video-recorded samples, and resolution of ambiguous criteria. A pre-test of 15 randomly selected student performances showed excellent inter-rater agreement for total OSCE scores (intraclass correlation coefficient = 0.87, 95% CI: 0.74–0.94). During formal assessments, brief debriefings after each rotation addressed scoring discrepancies to maintain consistency. Additionally, internal consistency for the 4 stations was good (Cronbach’s *α* = 0.82), indicating homogeneity across stations.

### Data analysis

All statistical analyses were carried out using SPSS 25.0. The test scores are expressed as the means ± SD and analyzed by an independent samples t-test, with Cohen’s d reported as the effect size (interpreted as small: *d* = 0.2, medium: *d* = 0.5, large: *d* = 0.8). Categorical data were analyzed by the chi-square test, with Cramer’s V reported for effect size. All the questionnaire data were analyzed using the Mann–Whitney U test, as these Likert-scale data were ordinal and failed to meet the normality assumption (Shapiro–Wilk test, all *p* < 0.05), with r for effect size calculated for interpretation (small: *r* = 0.1, medium: *r* = 0.3, large: *r* = 0.5). For significant group differences, 95% confidence intervals for the mean differences were also reported. For multiple comparisons (including 11 questionnaire items and four OSCE stations), we performed Bonferroni correction to adjust for Type I error inflation. The corrected significance level was set at *α* = 0.05/15 = 0.003. Statistical significance was defined as *p* < 0.05.

## Results

### Confidence of EBM-BOPPPS group in evidence-based medicine principles concept

**Figure 2:**
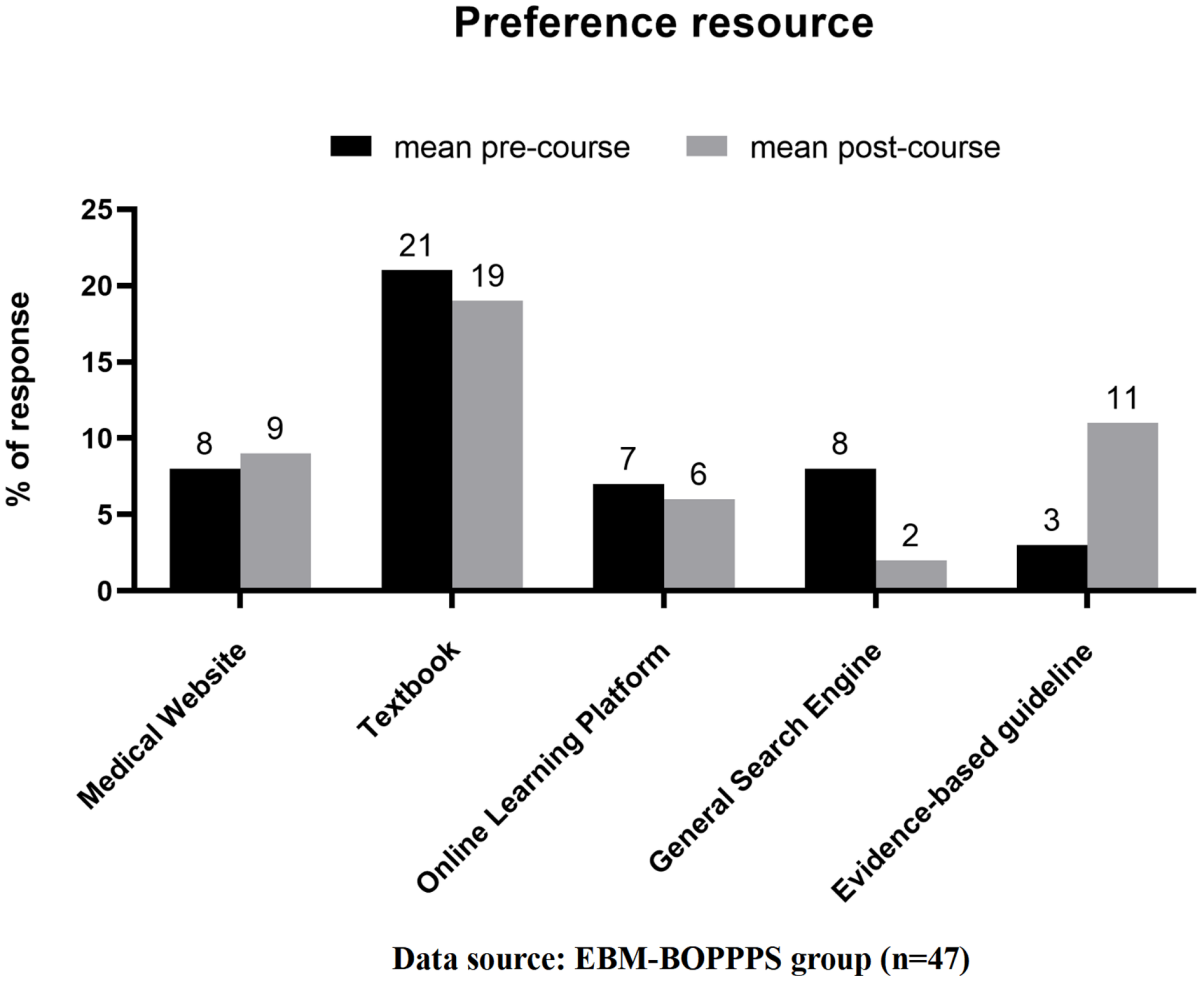
Changes in preferred learning resources of students in the EBM-BOPPPS group before and after the neurology rotation. Changes in preferred learning resources of students in the EBM-BOPPPS group before and after the neurology rotation. Data are presented as percentages of students preferring each resource type (textbook, evidence-based guideline, medical website, online learning platform, general search engine). Key shifts include a 3.8-fold increase in evidence-based guideline preference (6.4% pre-rotation vs. 23.4% post-rotation) and a 74.7% reduction in general search engine preference (17.0% pre-rotation vs. 4.3% post-rotation).

**Figure 3:**
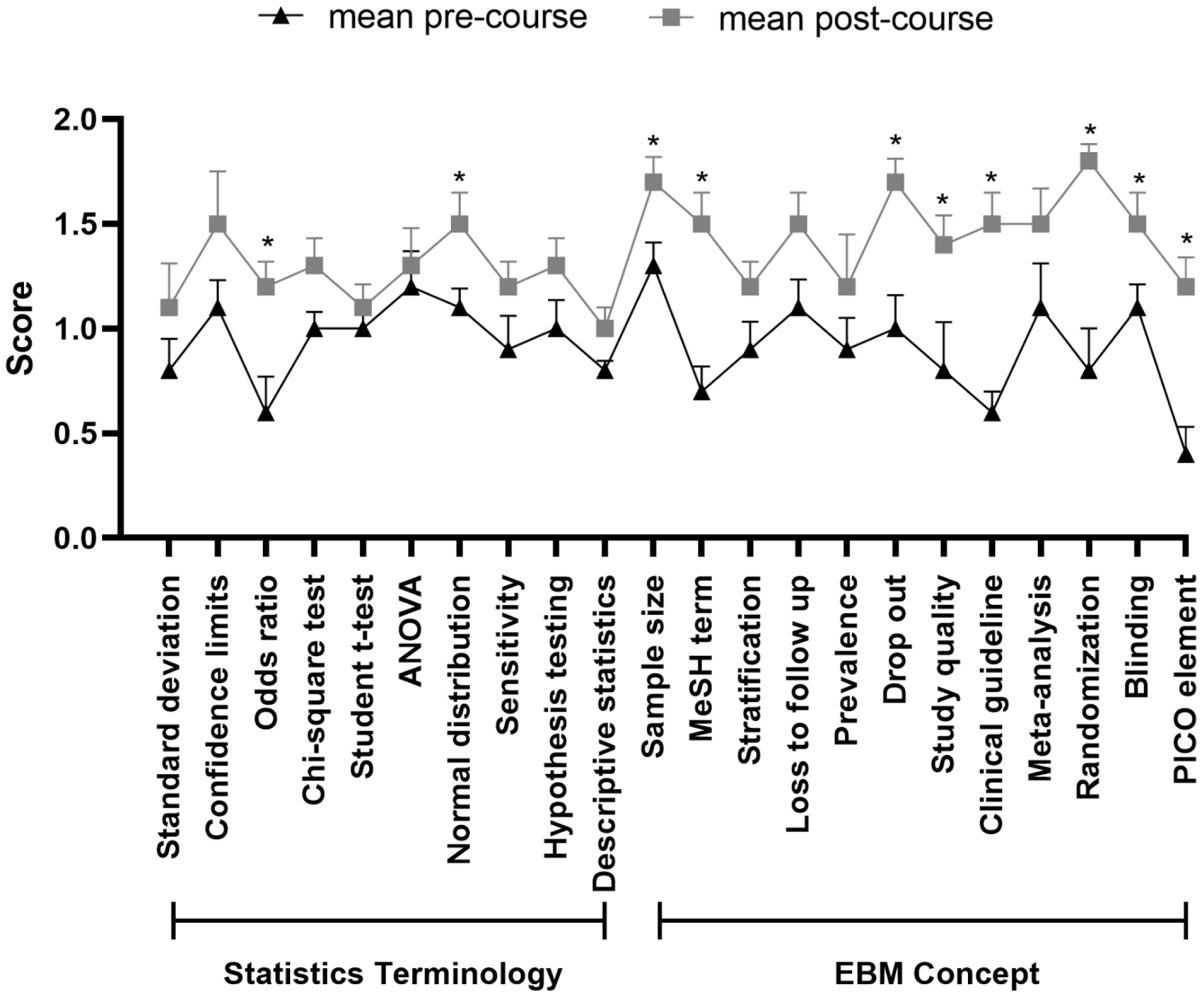
Pre- and post-rotation comparison of mean confidence scores for statistical terms and EBM concepts in the EBM-BOPPPS group. Pre- and post-rotation comparison of mean confidence scores for statistical terms and EBM concepts in the EBM-BOPPPS group. Confidence was scored on a 3-pomt scale (0 = no confidence, 1 = moderate confidence, 2 = high confidence) across 22 items (10 statistical terms, 12 EBM concepts; total possible score = 44). Total mean confidence scores increased from 20.1 ± 2.8 (pre-rotation) to 30.2 ± 3.3 (post-rotation); 8/12 EBM concepts and 2/10 statistical terms showed significant improvements (*p* < 0.05). The EBM Confidence Scale has confirmed psychometric properties: content validity index = 0.84, Cronbach’s *α* = 0.76. *indicates *p* < 0.05 (statistically significant difference).

At the beginning, students in the EBM-BOPPPS group exhibited a preference for utilizing study resources such as textbooks (44.7%), medical websites (17.0%), general search engines (17.0%), online learning platforms (14.9%), and evidence-based guidelines (6.4%). By the end of the rotation, the hierarchy of preferred study resources had shifted to prioritize textbooks (40.4%), followed by evidence-based guidelines (23.4%), medical websites (19.1%), online learning platforms (12.8%), and general search engines (4.3%) (Figure 2). Key shifts include a 3.8-fold increase in evidence-based guideline preference and a 74.7% reduction in general search engine preference. Furthermore, an analysis of mean pre-course scores revealed a general lack of confidence among students in their understanding of statistics terminology and concepts related to EBM, with the majority of students rating themselves as having low confidence levels in these areas. The pre-course total score, with a mean of 20.1 ± 2.8 out of a possible 44 points (based on 22 questions, each scoring a maximum of 2 points), showed a significant increase in confidence, as evidenced by the post-course total score mean of 30.2 ± 3.3. Notably, there was a marked improvement in the understanding of statistical concepts such as “odds ratio” and “normal distribution” compared to pre-course scores. Additionally, significant enhancements were observed in comprehension of EBM concepts, specifically in areas such as sample size, MeSH terms, drop out, study quality, clinical guidelines, randomization, blinding and PICO elements (Figure 3).

To further validate the scale’s construct validity, we analyzed the correlation between EBM Confidence Scale total scores and performance on the OSCE Physical Interview station (a key measure of EBM application). The EBM-BOPPPS group showed a moderate positive correlation between confidence scores and OSCE station scores (*r* = 0.35, *p* < 0.01), meaning students with higher self-reported EBM confidence were more likely to integrate EBM principles into clinical history-taking. This correlation supports that the scale captures a construct relevant to practical EBM skills.

### Comparison of the knowledge acquisition scores before and after the study

The pretest MCQs scores revealed no significant differences between the EBM-BOPPPS group and the control group, indicating comparable baseline medical knowledge (80.61 ± 7.60 vs. 81.22 ± 8.05, *p* = 0.703). Similarly, no significant differences were observed in the MCQs scores following the rotation (76.70 ± 6.85 vs. 77.02 ± 6.12, *p* = 0.809). However, post-intervention analysis showed that students in the EBM-BOPPPS group achieved significantly higher scores on the OSCE compared to the control (91.65 ± 2.54 vs. 88.86 ± 4.19, mean difference = 2.79, 95% CI: 1.30–4.21, Cohen’s *d* = 0.76, *p* < 0.001), which remained significant after Bonferroni correction for multiple comparisons (corrected *α* = 0.003). Specifically, the EBM-BOPPPS group outperformed the BOPPPS group in the physical interview station (20.82 ± 1.56 vs. 19.64 ± 1.78, mean difference = 1.18, 95% CI: 0.51–1.86, Cohen’s *d* = 0.71, *p* = 0.001), with significance retained after correction. Although the clinical judgment station showed a numerical advantage in the EBM-BOPPPS group (23.25 ± 1.49 vs. 22.44 ± 1.57, mean difference = 0.82, 95% CI: 0.19–1.43, Cohen’s *d* = 0.52, *p* = 0.011), this difference did not reach statistical significance after Bonferroni correction (*p* > 0.003). No significant differences were noted in the physical examination and communication stations (Table 2).

**Table 2:** Comparison of knowledge acquisition outcomes between EBM-BOPPPS and BOPPPS groups.

Group	Pre-rotation MCQs (100 points)	Post-rotation MCQs (100 points)	Post-rotation OSCE Stations (25 points/station)				Post-rotation OSCE Total (100 points)
			Station 1: Physical interview	Station 2: Physical examination	Station 3: Clinical judgment	Station 4: Communication skills	
EBM-BOPPPS (*n* = 47)	80.61 ± 7.60	76.70 ± 6.85	20.82 ± 1.56	22.04 ± 2.26	23.25 ± 1.49	25.53 ± 1.34	91.65 ± 2.54
BOPPPS (*n* = 50)	81.22 ± 8.05	77.02 ± 6.12	19.64 ± 1.78	21.70 ± 2.39	22.44 ± 1.57	25.08 ± 2.01	88.86 ± 4.19
*p* value	0.703	0.809	0.001*	0.471	0.011	0.201	0.000*
Mean Difference (95% CI)	−0.61 (−3.76 to 2.55)	−0.32 (−2.93 to 2.23)	1.18 (0.51–1.86)	0.34 (−0.59 to 1.28)	0.82 (0.19–1.43)	0.45 (−0.24 to 1.15)	2.79 (1.39–4.21)
Effect Size (Cohen’s *d*)	0.08	0.05	0.71	0.15	0.52	0.26	0.76

Effect size standards: Cohen’s *d* < 0.2 (small, negligible), 0.2–0.5 (small-to-medium), 0.5–0.8 (medium, educationally meaningful), >0.8 (large, highly meaningful). Medium-to-large effects indicate clinically relevant improvements. Statistically significant after Bonferroni correction (corrected *α* = 0.003).

*Denotes results with *p* < 0.003, indicating statistical significance after Bonferroni correction.

### Comparison of the satisfaction results between groups

Satisfaction with teaching was assessed using a 5-point Likert scale (1 = Strongly disagree to 5 = Strongly agree) and analyzed via the Mann–Whitney U test, with Bonferroni correction applied for multiple comparisons (corrected *α* = 0.003). see details in Supplementary Table 4.

In terms of learning attitude and general competencies (Table 3), the EBM-BOPPPS group showed more positive tendencies in several aspects compared to the BOPPPS group, though these differences did not reach statistical significance after Bonferroni correction (corrected *α* = 0.003). For learning attitude items, the EBM-BOPPPS group had a median score of 4 (IQR = 0) for both “It is easy to know the learning goals” (*U* = 886.2, *r* = 0.19, uncorrected *p* = 0.048) and “The course helps enhance my learning motivation” (*U* = 834, *r* = 0.24, uncorrected *p* = 0.020), while the BOPPPS group had a median of 4 (IQR = 0) and 4 (IQR = 1) respectively. Regarding general competencies, “The course develops my problem-solving skills” saw the EBM-BOPPPS group with a median of 4 (IQR = 0) and the BOPPPS group with 4 (IQR = 1) (*U* = 744, *r* = 0.31, uncorrected *p* = 0.003), and no significant differences were found in the other three general competency items, with all uncorrected *p* values far exceeding 0.003.

**Table 3:** Comparison of learning attitude and general competencies satisfaction between EBM-BOPPPS and BOPPPS groups.

Question category	Specific question	Group	Median (Interquartile Range)	Mann–Whitney U	Effect size (*r*)	*p* Value
Learning Attitude	It is easy to know the learning goals	EBM-BOPPPS	4 (0)	886.2	0.19	0.048
		BOPPPS	4 (0)			
	The course helps enhance my learning motivation	EBM-BOPPPS	4 (0)	834	0.24	0.020
		BOPPPS	4 (1)			
General Competencies	The course develops my problem-solving skills	EBM-BOPPPS	4 (0)	744	0.31	0.003*
		BOPPPS	4 (1)			
	The course promotes the memorization of knowledge	EBM-BOPPPS	4 (0)	994	0.05	0.610
		BOPPPS	4 (1)			
	The course improves my communication skills	EBM-BOPPPS	4 (0)	1,017	0.03	0.780
		BOPPPS	4 (0)			
	The course improves my ability to give presentations	EBM-BOPPPS	4 (0)	927	0.01	0.920
		BOPPPS	4 (0)			

Likert scale: 1 = Strongly disagree, 2 = Disagree, 3 = Neutral, 4 = Agree, 5 = Strongly agree. All statistics use Mann–Whitney U test (non-parametric). Bonferroni-corrected *α* = 0.003.

*Denotes results with *p* < 0.003, indicating statistical significance after Bonferroni correction.

For EBM-specific skills (Table 4), the EBM-BOPPPS group exhibited significantly higher satisfaction than the BOPPPS group, and all key differences remained significant after Bonferroni correction. In “I can formulate a clinical question to search the best evidence,” the EBM-BOPPPS group had a median of 4 (IQR = 1) versus 3 (IQR = 1) in the BOPPPS group (*U* = 715, *r* = 0.43, uncorrected *p* = 0.001). “I am confident in critically appraising a journal article” showed a median of 4 (IQR = 0) in the EBM-BOPPPS group and 3 (IQR = 1) in the BOPPPS group (*U* = 781, *r* = 0.30, uncorrected *p* = 0.003). Additionally, “I consider evidence-based medicine important to my future career” had a median of 4 (IQR = 1) in the EBM-BOPPPS group compared to 3 (IQR = 1) in the BOPPPS group (*U* = 699, *r* = 0.43, uncorrected *p* = 0.001), all meeting the corrected significance standard.

**Table 4:** Comparison of EBM-specific skills satisfaction between EBM-BOPPPS and BOPPPS groups.

Question category	Specific question	Group	Median (Interquartile Range)	Mann–Whitney U	Effect size (*r*)	*p* Value
EBM-Specific Skills	I can formulate a clinical question to search the best evidence	EBM-BOPPPS	4 (1)	715	0.43	0.001*
		BOPPPS	3 (1)			
	I am confident in critically appraising a journal article	EBM-BOPPPS	4 (0)	781	0.30	0.003*
		BOPPPS	3 (1)			
	I consider evidence-based medicine important to my future career	EBM-BOPPPS	4 (1)	699	0.43	0.001*
		BOPPPS	3 (1)			

Likert scale: 1 = Strongly disagree, 2 = Disagree, 3 = Neutral, 4 = Agree, 5 = Strongly agree. All statistics use Mann–Whitney U test (non-parametric). Bonferroni-corrected *α* = 0.003.

*Denotes results with *p* < 0.003, indicating statistical significance after Bonferroni correction.

Regarding learning burden and stress (Table 5), the EBM-BOPPPS group reported higher perceived burden and stress, but these differences were not significant after Bonferroni correction. “I consider this course taking up too much of my preparation time” had a median of 3 (IQR = 1) in the EBM-BOPPPS group and 2 (IQR = 1) in the BOPPPS group (*U* = 792, *r* = 0.19, uncorrected *p* = 0.064). “I consider the preparation and presentation for this course is quite stressful for me” showed a median of 3 (IQR = 0) in the EBM-BOPPPS group versus 2 (IQR = 1) in the BOPPPS group (*U* = 877, *r* = 0.23, uncorrected *p* = 0.026). Furthermore, there are 4 students on the online platform who left messages such as “the course is too difficult to keep up with,” “cannot understand the literature,” “do not like this form of teaching,” and “need to stay up late to prepare assignment.”

**Table 5:** Comparison of learning burden and stress satisfaction between EBM-BOPPPS and BOPPPS groups.

Question category	Specific question	Group	Median (Interquartile Range)	Mann–Whitney U	Effect size (*r*)	*p* Value
Learning Burden & Stress	I consider this course taking up too much of my preparation time	EBM-BOPPPS	3 (1)	792	0.19	0.064
		BOPPPS	2 (1)			
	I consider the preparation and presentation for this course is quite stressful for me	EBM-BOPPPS	3 (0)	877	0.23	0.026
		BOPPPS	2 (1)			

Likert scale: 1 = Strongly disagree, 2 = Disagree, 3 = Neutral, 4 = Agree, 5 = Strongly agree. All statistics use Mann–Whitney U test (non-parametric). Bonferroni-corrected *α* = 0.003.

To explore the potential impact of digital literacy on EBM skill outcomes, we conducted a post-hoc analysis of the four students who reported “difficulty navigating databases” (all from the EBM-BOPPPS group), comparing their EBM-related performance to the remaining 43 students in the same group. The four students showed smaller pre-post improvements in the digital literacy-relevant item in the EBM Confidence Scale: “MeSH term” (mean increase: 0.3 ± 0.5 vs. 0.8 ± 0.4, *p* = 0.032), a finding consistent with their reported database navigation challenges, as MeSH terms are core tools for efficient medical literature retrieval. These four students had numerically lower total EBM Confidence Scale scores (21.5 ± 1.2 vs. 23.4 ± 1.5, *p* = 0.061), but the difference did not reach statistical significance, indicating that digital literacy difficulty had limited impact on overall EBM application ability. Moreover, no significant differences were observed in EBM Confidence Scale items related to “formulating clinical questions” (PICO element, *p* = 0.124) or “critically appraising journal articles” (Study quality, *p* = 0.187), suggesting that the EBM-BOPPPS intervention’s structured support mitigated the impact of digital literacy gaps.

To further validate whether the observed improvements in study-specific outcomes were driven by the EBM-BOPPPS intervention rather than the Hawthorne effect, we conducted a supplementary analysis of participants’ performance in a non-study-related Internal Medicine Clerkship Assessment. This assessment is defined as the average score of end-of-rotation evaluations for internal medicine subspecialties including endocrinology, cardiology, and gastroenterology which completed by all participants before the neurology clerkship. This assessment focuses on common internal medicine conditions with no overlap with neurology or EBM content, making it a reliable proxy for routine academic performance unaffected by our intervention.

As shown in Table 6, both groups exhibited small but statistically significant improvements in internal medicine scores from pre- to post-intervention—consistent with the expected skill progression during clinical internships: the EBM-BOPPPS group increased from 75.2 ± 5.4 to 77.8 ± 4.9 (*p* = 0.042), and the BOPPPS group increased from 76.1 ± 5.1 to 78.5 ± 4.8 (*p* = 0.038). Critically, there were no significant differences in baseline scores (*p* = 0.516, Cohen’s *d* = 0.17) or post-intervention scores (*p* = 0.721, Cohen’s *d* = 0.14) between the two groups, and their average improvement magnitudes were nearly identical (2.6 points vs. 2.4 points). This pattern confirms that the score improvements were due to routine internship skill development, as both groups benefited equally, this ruling out the possibility that the EBM-BOPPPS group’s study-specific gains were driven by the Hawthorne effect.

**Table 6:** Comparison of non-study-related internal medicine clerkship assessment scores between EBM-BOPPPS and BOPPPS groups.

Variable	EBM-BOPPPS group (*n* = 47)	BOPPPS group (*n* = 50)	Between-group *p* value	Effect size (Cohen’s *d*)
Pre-intervention internal medicine score	75.2 ± 5.4	76.1 ± 5.1	0.516	0.17
Post-intervention internal medicine score	77.8 ± 4.9	78.5 ± 4.8	0.721	0.14
Within-group *p* value	0.042	0.038	–	–
Average improvement (post-pre, points)	2.6	2.4	–	–

Post-intervention scores reflect end-of-study re-evaluation of the same subspecialties, consistent with the internship assessment system (small score increases align with expected clinical skill progression). Statistical methods: Independent samples t-test for between-group comparisons, paired samples t-test for within-group comparisons; Cohen’s *d* < 0.2 indicates a negligible effect size.

## Discussion

### Introducing EBM to medical clerks through the EBM-BOPPPS method is meaningful

Integrating Evidence-Based Medicine into clinical practice has become a cornerstone of modern healthcare, yet determining the optimal stage to introduce it to medical clerks remains a complex challenge (21, 22). EBM places emphasis on applying up-to-date research findings, encouraging students to take an active role in learning while enhancing clinical decision-making through practitioner insights. This stands in contrast to traditional medical teaching methods, which often fall short of meeting the evolving demands of contemporary clinical education (23). A study exploring junior doctors’ understanding and attitudes toward EBM found that although they recognized the value of EBM skills in clinical work, their lack of sufficient training left them feeling unconfident in applying these skills (24, 25). Medical students lack knowledge and skills related to EBM and have a positive attitude toward its use in healthcare practice (26).

This study illustrates that the integration of EBM into the curriculum for medical clerks, utilizing the BOPPPS instructional model, is well-received by students and significantly enhances their competency in EBM during the clerkship phase. The field of neurology is characterized by diverse diagnostic criteria and clinical manifestations, and clinical guidelines grounded in EBM are crucial for the accurate diagnosis and treatment of neurological disorders (20, 27). Traditional lecture-based neurology education has historically prioritized theoretical knowledge over practical application, rote memorization over critical thinking, and grade attainment over the development of individual competencies, as previously documented (28). Studies have explored various approaches to improve neurology education, including the use of technology (29), structured clinical experiences (30), and evidence-based guidelines (31). However, limited research has specifically addressed EBM teaching within neurology clerkships. Our study indicates that the integration of EBM with the BOPPPS instructional model is more effective in enabling medical clerks to achieve the intended educational objectives in neurology education.

### The EBM-BOPPPS model was effective in enhancing students’ awareness and utilization of evidence-based principles

Prior to the clerkship, a small percentage of students in the EBM-BOPPPS group favored evidence-based resources as their primary source of information to support their clinical questions. Students usually rely on instant tools and search engines, and rarely use academic databases such as PubMed. This may be due to the lack of proper systematic training, insufficient reinforcement of usage scenarios, and inadequate awareness of evidence-based medicine, which is consistent with the conclusions of previous studies (32). However, after completing the neurology clerkship, there was a significant increase in the number of students who rated evidence-based resources as their first choice in experimental group. Additionally, there was a notable decrease in the number of students who preferred general search engines.

Integrated application of diverse teaching strategies can more significantly enhance students’ EBM skills and improve their attitudes toward evidence-based practice, rather than simply introduce the concepts to memorize (22, 33, 34). Although students have learned statistical terms in epidemiology courses before neurology clerkship, they did not demonstrate significant improvement in their understanding of statistical terminology before or after class. Out of 10 terms assessed, only 2 showed improved scores, potentially due to the insufficient medical statistics training of medical clerks as previously noted (35). In contrast, out of the 12 terms related to EBM, 8 showed significant improvement in scores, suggesting an increased familiarity with the concept of EBM among students. Following the clerkship, students’ attitudes toward EBM improved, as evidenced by their increased confidence in developing problem-solving skills, searching for evidence, critically appraising journal articles, and recognizing the importance of EBM in their future careers.

This progress was measured using a 22-item EBM Confidence Scale, which itself addresses critical gaps in EBM assessment for Chinese junior college clerks. Developed via a composite framework which blend international EBM core principles, Chinese grassroots clinical priorities, and national three-year college medical education standards, the scale is closely tailored to the group’s competency needs. Its strong psychometric rigor ensures it is a trustworthy tool for evaluating EBM confidence in this population. Importantly, the scale fills a gap identified in prior systematic reviews. Existing EBM assessment tools target five-year undergraduates or postgraduates, include advanced content irrelevant to three-year clerks, and lack grassroots focus (21). Our scale resolves this by excluding rarely used advanced content, adding grassroots-specific items, and adapting evidence appraisal to the clerks’ EBM foundation. Further, amid the absence of official EBM competency guidelines for Chinese junior college clerks, the composite framework offers a reusable “guideline alternative” and can be adapted to evaluate EBM skills in other grassroots-focused groups.

#### The EBM-BOPPPS teaching model helps improve students’ knowledge acquisition compared to BOPPPS control

A key observation was the intervention’s differential effect on outcomes: significant OSCE improvements (Cohen’s *d* = 0.76, *p* < 0.001) but non-significant MCQ performance (*p* = 0.38). This pattern is explained by the distinct focus of each outcome measure. The MCQ specifically evaluated foundational neurological knowledge, of which the content is covered in pre-clerkship core courses such as Neurology and Pathophysiology and is mastered by all students before the rotation. The lack of significant variance in MCQs scores between the two groups may be attributed to the fact that MCQs essentially examine knowledge memory and ability (36), while the “Pre-assessment” and “Post-assessment” sections of BOPPPS can ensure that both groups of students have the same level of mastery of basic knowledge through repeated reinforcement.

In contrast, the OSCE measured the ability to apply knowledge to clinical scenarios. Under the modified OSCE test for junior-level medical clerks, integrating EBM-BOPPPS significantly improved students’ total OSCE scores, with robust significance retained after Bonferroni correction. The 2.79-point total OSCE gain and 1.18-point Physical Interview gain have tangible clinical and educational value: for example, accurate capture of “abrupt onset” and “atrial fibrillation history” in ischemic stroke helps avoid delays in thrombolysis or incorrect antiplatelet selection, which is critical for reducing stroke morbidity. Although the Clinical Judgment station showed a numerical advantage with a moderate effect size (Cohen’s *d* = 0.52), this difference did not reach statistical significance after correction, suggesting the need for larger sample sizes to confirm this trend. The total score improvement thus primarily stems from the history-taking component and overall clinical integration, rather than all individual stations.

Notably, for three-year junior college medical clerks targeted for grassroots primary care, this OSCE improvement which focused on application, is particularly valuable. Grassroots clinics prioritize “using guidelines to manage common neurology cases” over “recalling basic neurology theories,” and the intervention’s ability to improve OSCE (application) but not MCQ (pre-mastered basics) aligns with their future clinical needs, bridging the education-practice gap by turning statistical improvements into skills that directly address grassroots clinics’ need for safe, evidence-based care.

#### The EBM-BOPPPS teaching model fostered a positive learning attitude compared to BOPPPS control

The BOPPPS approach is being embraced by universities around the world. Our adaptation of the BOPPPS framework focuses on both solo and collaborative involvement, utilizing evidence-based reasoning to solve clinical problems. As previously noted (37), EBM education is often viewed as tedious and not very stimulating, particularly in the area of neurology. Nevertheless, the BOPPPS method improves engagement and accessibility.

Students in the EBM-BOPPPS cohort reported heightened satisfaction across two primary dimensions, with the results maintaining statistical significance following Bonferroni correction. Firstly, regarding EBM-specific skills, the cohort exhibited strong significance in core competencies, including the ability to formulate clinical questions, confidence in critically appraising journal articles, and recognition of the importance of EBM in their future careers. These outcomes are consistent with the model’s focus on evidence-based reasoning, directly affirming its role in cultivating essential EBM skills, in alignment with the intervention’s design as an EBM-integrated framework. Secondly, in terms of general competencies, the EBM-BOPPPS cohort also demonstrated a statistically significant enhancement in “problem-solving skills” (post-correction), a critical practical ability for clinical practice. This finding indicates an extension of the model’s impact beyond EBM-specific training to the development of fundamental clinical skills, illustrating how the interactive, learner-centered BOPPPS structure if combined with EBM’s systematic reasoning training, promotes not only evidence-based thinking but also practical problem-solving abilities. While trends of enhanced learning motivation and clearer learning objectives were observed in the EBM-BOPPPS group with small-to-moderate effect sizes, these did not achieve statistical significance after correction.

While the EBM-BOPPPS model improved clinical and EBM competencies, the higher perceived stress and preparation burden warrant attention. Qualitative feedback that four student comments noting difficulty with literature and late-night preparation suggests this stress stems from two factor. Firstly, the novel EBM tasks requiring self-directed learning, consistent with Shahrani et al. (22), who reported junior students often find EBM skill acquisition initially demanding. Moreover, the three-year college program’s compressed timeline, where clerks balance multiple rotations. This finding is not unique to our study, Woezik et al. (34) also observed increased workload perception with practice-based EBM teaching, but noted stress diminished with repeated training.

### Limitations and further improvements

#### Statistical analysis considerations

The Bonferroni correction, while reducing Type I error, may increase Type II error in exploratory analyses with small to moderate effect sizes. However, to address concerns about false positives, we supplemented Bonferroni-corrected results, which confirmed that the core findings including total OSCE scores, physical interview performance, confidence in problem-solving skills, and recognition of EBM’s professional importance, remained statistically significant. This robustness strengthens our conclusion that the EBM-BOPPPS model effectively enhances key clinical and EBM competencies. For outcomes with moderate effect sizes but non-significant correction results such as clinical judgment station and problem-solving abilities, we interpret them as exploratory trends that warrant further validation in larger cohorts, rather than definitive conclusions.

#### Potential Hawthorne effect

As this study involved active participation of students in structured teaching interventions, the Hawthorne effect may have influenced outcomes. However, supplementary analysis of the Internal Medicine Clerkship Assessment provides evidence that the Hawthorne effect had minimal impact on core findings. This assessment which was defined as the average score of end-of-rotation evaluations for endocrinology, cardiology, and gastroenterology (all completed before the neurology clerkship) and its post-intervention re-evaluation (conducted after the neurology clerkship but focusing on the same internal medicine subspecialties) showed that both groups exhibited small but statistically significant score improvements, consistent with routine internship skill progression. Critically, there were no significant between-group differences in baseline scores (*p* = 0.516), post-intervention scores (*p* = 0.721), or improvement magnitudes (EBM-BOPPPS: +2.6 points vs. BOPPPS: +2.4 points, *p* = 0.892). This confirms that the score gains were driven by regular clinical training (not generalized attention from study participation), as this internal medicine assessment focused on non-neurology, non-EBM content and was unrelated to the EBM-BOPPPS intervention in the neurology clerkship. In contrast, the EBM-BOPPPS group’s significant advantages in study-specific outcomes are unlikely to be explained by the Hawthorne effect, as such gains were not observed in this non-study-related internal medicine performance.

#### Short-term assessment and lack of long-term follow-up

All outcome assessments, such as the modified OSCE, student satisfaction surveys, and MCQs assessing basic neurological knowledge, were conducted right after the intervention period, aligning with the conclusion of the two-month neurology clerkship. The timing indicates that the outcomes mainly capture short-term learning effects, like the immediate recall of EBM concepts or temporary enhancements in clinical skills shown during the post-clerkship evaluation. They fail to measure long-term knowledge retention and do not determine if improvements in EBM skills or clinical competence are sustainable. Without long-term data, we cannot fully confirm the durability of the intervention’s effects, which is a key consideration for evaluating the practical value of the EBM-integrated BOPPPS model in supporting long-term clinical skill development for junior college medical clerks.

Nevertheless, the immediate improvements observed in this study provide indirect support for the potential sustainability of intervention effects, particularly for the grassroots medical clerk population. First, the EBM-BOPPPS group showed significant pre-post improvements in 8 out of 12 EBM-related concepts, with a mean total EBM confidence score increase of 10.1 points. Previous studies have confirmed that mastery of EBM core concepts is a strong predictor of long-term skill retention and this foundational understanding reduces the risk of rapid skill decay and provides a cognitive framework for subsequent practical application in grassroots clinics (38). Secondly, the marked enhancement observed in the OSCE Physical Interview station corresponds with the clinical skill most commonly employed in primary care settings. The repeated application of this EBM integrated skill in routine practice may reinforce habit formation, thereby potentially mitigating the decline of improvements induced by the intervention.

#### Time burden on instructors

Beyond initial training, instructors spent up to 2 extra hours per week preparing EBM case materials compared to the standalone BOPPPS model. Task-based logging shows the extra time averaged 1.8 ± 0.4 h/week and this burden is feasible for grassroots hospital instructors, as contextualized by their actual workload: the four participating instructors had a weekly total teaching workload of 5–6 h including clerkship supervision, lectures, and case discussions, meaning the extra EBM preparation accounted for ~30% of their weekly teaching time. Additionally, a 20% reduction in extra time to 1.4 ± 0.3 h/week was observed in the later intervention stages (Weeks 6–8) as instructors reused and adapted existing EBM materials, indicating potential for burden reduction with standardization.

#### Unaddressed student baseline variability

While we stratified randomization by gender and epidemiology scores, we did not account for variability in digital literacy which is critical for EBM literature search, as observed in four student feedback comments noting difficulty navigating databases.

However, several factors limit the impact of this unaddressed variable on our core conclusions. First, all participants completed a literature search module in their pre-rotation Epidemiology course, ensuring minimal baseline digital literacy proficiency and narrowing potential between-group disparities. Second, post-hoc analysis of the four students with database navigation difficulties showed that their digital literacy gaps only affected “database-specific confidence” but not overall EBM skills. Third, the EBM-BOPPPS intervention’s design including pre-curated evidence resources and step-by-step database tutorials in the 2-h offline training, which reduced reliance on independent digital literacy, further mitigating the influence of baseline variability. These factors collectively suggest that digital literacy did not serve as a major confounding variable distorting the intervention’s effect on core EBM competencies.

#### EBM confidence scale validation considerations

While the initial 22-item 3-point EBM Confidence Scale was not a pre-validated standardized tool, supplementary analyses addressed this limitation: content validity (CVI = 0.84) confirmed item relevance to grassroots clerk competencies, internal consistency (Cronbach’s *α* = 0.76) ensured reliable measurement, and correlation with OSCE Physical Interview scores (*r* = 0.35, *p* < 0.01) demonstrated alignment with objective EBM practice. A systematic review by Kumaravel (21) notes that no standardized EBM confidence scale exists for three-year junior college clerks and our scale was tailored to this gap, excluding advanced terms irrelevant to grassroots practice and focusing on applicable concepts. This targeted design, paired with validation, mitigates concerns about measurement reliability.

#### Future improvement directions

To improve the EBM-BOPPPS model, future research should focus on six areas. Firstly, including a control group receiving standard standalone BOPPPS teaching to measure the Hawthorne effect and better attribute outcomes to the EBM-integrated model. Secondly, conducting structured long-term follow-ups of the current cohort to assess lasting knowledge and skill retention: we will conduct two rounds of assessments at 6 and 12 months post-clerkship, with the first using a scenario-based EBM test adapted from this study’s modified OSCE criteria ensuring consistency with baseline post-intervention outcomes and the second employing a supervisor rating scale completed by grassroots preceptors measuring EBM application frequency in real consultations. Currently, we have retained contact information for 92% of participants (43/47 in the EBM-BOPPPS group, 49/50 in the control group) and submitted a follow-up study application linked to the current teaching reform project. Thirdly, enhancing scalability and workload evidence through two measures: (1) Creating standardized EBM teaching toolkits and offline resources to ease instructor workload and ensure consistent application across educational settings; (2) Implementing systematic instructor time-tracking using the Workload Assessment for Teaching Staff, which records teaching-related time in real time, distinguishes between “one-time preparation” and “recurring preparation,” and collects instructor-perceived workload via a 5-point Likert scale (1 = No burden to 5 = Severe burden), providing robust data on time burden across different grassroots institutions. Fourthly, incorporating a validated digital literacy baseline assessment before intervention implementation, drawing on students’ existing foundation from the Epidemiology course’s literature search module. We will stratify participants by digital literacy level during randomization to balance this variable across groups, and provide targeted pre-intervention digital literacy workshops for students with low baseline skills, ensuring EBM skill development is not limited by database navigation barriers. Fifthly, collaborating with EBM education experts to expand the current 22-item scale into a validated tool specifically for grassroots medical clerks incorporating feedback from this study. Using Perraton’s longitudinal framework (14), we will test the expanded scale’s test–retest reliability and criterion validity, ensuring it meets international validation standards for medical education tools.

#### Generalizability considerations

This study was conducted in a Chinese three-year junior college medical program, with participants targeted for primary care roles in grassroots health facilities, which limits direct generalizability to other settings. The EBM-BOPPPS model’s design which include the 2-h basic EBM workshops and 2-month condensed neurology clerkship aligned with the program’s focus on practical, short-term training, making it less applicable to five-year undergraduate or postgraduate programs that require advanced EBM skills. Additionally, as noted in limitations, the intervention relied on online platforms and paid databases, restricting scalability to resource-constrained rural colleges or low- and middle-income countries without offline adaptations. While the model may be conditional for similar primary care-oriented short-duration programs or other Chinese three-year junior college clerkships, given consistent student baseline and teacher-led norms, future studies should test it in diverse settings with context-specific adjustments to validate broader applicability.

## Conclusion

The EBM-BOPPPS teaching model incorporates the core principles of EBM and encourages three-year junior college medical clerks to independently identify and appraise academic resources. This strategy converts passive information gathering into active knowledge creation by embedding systematic literature searches within organized learning steps. Engaging students enhances their understanding of clinical reasoning and develops the critical evaluation skills needed for evidence-based practice. The BOPPPS model provides a structured framework for interactive learning, with EBM principles enhancing rigor and relevance at each step. This method boosts educational achievements beyond just memorizing, empowering learners to analyze clinical problems, use high-quality evidence, and personalize interventions based on patient preferences. This alignment of instructional design with real-world clinical reasoning positions the EBM-BOPPPS approach as a transformative strategy for preparing future grassroots healthcare professionals to navigate evolving medical landscapes.

## Data Availability

The raw data supporting the conclusions of this article will be made available by the authors without undue reservation.
