# Supplementary material for: Effect of the EBM-integrated BOPPPS model on clinical competence and EBM confidence in neurology clerkships for three-year junior college medical clerks
Source: Front Public Health. 2025 Oct 13;13:1676073. doi: 10.3389/fpubh.2025.1676073 (PMC12554736; doi:10.3389/fpubh.2025.1676073)
Supplement: Supplementary file 1 [file Data_Sheet_1.PDF]

Supplemental table 1

EBM-BOPPPS teaching process illustrated with *Ischemic stroke*

EBM-BOPPPS	Contents
**B**ridge-in	**Case:** A 65-year-old male patient was admitted to the hospital in June 2023. Approximately two hours prior to breakfast, he experienced an abrupt onset of right-sided limb weakness and dysarthria, with no accompanying headache, emesis, or altered consciousness. Neurological assessment revealed a diminished right nasolabial fold, muscle strength graded at 3 in the right limb, and a positive Babinski sign on the right side. Physical examination: T 36.8℃, P 98/min, R 20/min, BP 150/90mmHg, BMI 28.<br>**Question 1:** What immediate diagnostic tests should be ordered for this patient?<br>**Question 2**: Is the patient eligible for intravenous thrombolysis?<br>**Question 3**: Which antiplatelet medication is most appropriate for secondary prevention in this patient?<br>**Question 4**: What is the recommended target value for blood pressure reduction?<br>**Question 5**: Suppose he has a history of atrial fibrillation. How would this alter your diagnostic workup and acute treatment priorities?
**O**bjectives	Students were expected to master the definition, etiology, clinical manifestations and therapeutic principles of *ischemic stroke*.
**P**re-assessment *(5 min)*	Students completed the assessment test online prior to engaging in the class.
**P**articipatory learning *(35 min)*	1) Students engaged in group discussions regarding the case for analysis. They participated in evidence-based decision-making exercises that addressed the proposed questions, utilizing retrieval tools to access the most recent guidelines..<br>2) Each group appointed a representative to present their discussion findings, detailing the analytical methods employed and the treatment strategies developed.<br>3) The instructor consolidated and evaluated the research evidence and remedial measures proposed by all groups, thoroughly addressing the inquiries raised in the case.<br>4) The instructor summarized and presented the evidence-based approach to formulating questions, retrieving evidence, and applying that evidence.
**P**ost-assessment *(5 min)*	The instructor assessed the students' comprehension of the course through the completion of online test at the conclusion of the class.
**S**ummary *(5min)*	The instructor summarized the etiology, clinical manifestations, diagnosis, treatment, and prognosis of ischemic infarction.

Supplemental table 2

Composite Framework for Content Validity Evaluation of the 22-Item EBM Confidence Scale

Framework Component	Allocated Scale Item	Item Number	Rationale for Allocation
1. EBM Core Principles (Kumaravel et al., 2021)	PICO element	22	Directly aligns with the "Ask" step of the 5-step EBM process (Ask→Acquire→Appraise→Apply→Assess), which is the starting point for EBM practice and a core part of EBM core principles.
	MeSH term	12	Corresponds to the "Acquire" step of the 5-step EBM process, as MeSH terms are key tools for efficient evidence retrieval, a fundamental skill in EBM core principles.
	Study quality	17	Matches the "Appraise" step of the 5-tep EBM process, which is essential for judging the reliability of evidence, a core component of EBM core principles.
	Clinical guideline	18	Aligns with the "Apply" step of the 5-step EBM process, as clinical guidelines are the main form of applying evidence in clinical practice, a key part of EBM core principles.
	Loss to follow - up	14	Relates to the "Assess" step of the 5-step EBM process, as loss to follow - up is a critical factor in evaluating the validity of evidence application outcomes, part of EBM core principles.

	Randomization	20	A foundational concept in EBM study design, which is a key part of EBM core knowledge under EBM core principles.
	Blinding	21	An important aspect of EBM study design to reduce bias, belonging to EBM core knowledge in EBM core principles.
2. Clinical Needs of Chinese Grassroots Hospitals	Prevalence	15	Prevalence is widely used in grassroots hospitals to assess disease burden and plan screening strategies, directly meeting grassroots clinical needs.
	Sample size	11	Grassroots hospitals often conduct small - scale observational studies; understanding sample size is essential for ensuring research validity, fitting grassroots clinical needs.
	Dropout	16	High dropout rates are common in grassroots patient management; this item targets the practical need to maintain data integrity, aligning with grassroots clinical needs.
	Stratification	13	Grassroots populations have diverse characteristics; stratification helps improve the accuracy of clinical decision - making for specific groups, meeting grassroots clinical needs.
3. The Teaching Standards for Three-Year College Clinical	Standard deviation	1	Explicitly listed as basic statistical knowledge in the national teaching standards, a mandatory part of the curriculum.
	Confidence limits	2	Required basic statistical content in the national teaching standards for three - year college clinical medicine programs.

Medicine Programs (2023)	Odds ratio	3	Included in the statistical knowledge module of the national teaching standards, a core mandatory term.
	Chi - square test	4	A basic statistical method required by the national teaching standards for clinical data analysis.
	Student's t - test	5	Explicitly mandated in the national teaching standards as a foundational statistical skill.
	ANOVA	6	Included in the intermediate statistical knowledge section of the national teaching standards.
	Normal distribution	7	A basic statistical concept required by the national teaching standards for understanding clinical data distribution.
	Sensitivity	8	Listed in the national teaching standards as key knowledge for evaluating diagnostic test performance.
	Hypothesis testing	9	A foundational statistical principle required by the national teaching standards for evidence - based thinking.
	Descriptive statistics	10	The most basic statistical skill mandated by the national teaching standards for clinical data presentation.
	Meta-analysis	19	Included in the EBM knowledge module of the national teaching standards as an introductory EBM concept.

Supplemental table 3

The modified OSCE form for clerks in Neurology Department

Stations	Content	Assessment Criteria & Scoring (10 points each station)	Score
**1. Physical Interview** ***(8 min)*** *Dr. Xiao*	***Complete history taking*** ***（0-25points）***	1. Onset inquiry (5 points): Accurately obtain onset time, mode (sudden/gradual), and key symptoms<br>2. Associated symptoms (5 points): Targeted inquiry about neuro-related symptoms<br>3. Past history & risk factors (5 points): Ask about hypertension, diabetes, atrial fibrillation, and prior similar episodes<br>4. Logic & efficiency (5 points)<br>5. Communication attitude (5 points)	
**2. Physical Examination** ***(8 min)*** *Dr. Lang*	***Perform targeted neurological exams based on Station 1 history*** ***（0-25points）***	1. Relevance (8 points): Focus on key signs<br>2. Technical correctness (8 points): Proper techniques and logical order<br>3. Result accuracy (5 points): Precisely describe positive/negative signs<br>4. Patient comfort (4 points): Gentle movements, explain purposes, avoid unnecessary exposure	
**3. Clinical Judgment** ***(5 min)*** *Dr. Gu*	***Make diagnosis and differential diagnosis using history, exam, imaging/lab results*** ***（0-25points）***	1. Primary diagnosis (5 points): Clear and consistent with case features<br>2. Differential diagnosis (5 points): List relevant differential diagnoses<br>3. Result interpretation (8 points): Correctly interpret imaging and labs<br>4. Next test suggestions (7 points): Reasonable recommendations	
**4. Communication Skills** ***(5 min)*** *Dr. Zhang*	***Communicate in scenarios (condition briefing and patient education)*** ***（0-25points）***	1. Information accuracy (10 points): Correct medical content<br>2. Clarity (8 points): Use plain language, avoid jargon, ensure understanding<br>3. Empathy & response (7 points): Acknowledge emotions, address questions effectively	
**Total**			

Supplemental table 4

Students' perspectives on EBM-BOPPPS vs BOPPPS teaching modes

Questions	Group	Strongly disagree n (%)	Disagree n (%)	Neutral n (%)	Agree n (%)	Strongly agree n (%)
**It is easy to know the learning goals**	**EBM-BOPPPS**	0 (0%)	1 (2.1%)	5 (10.6%)	30 (63.8%)	11 (23.4%)
	**BOPPPS**	1 (2%)	4 (8%)	10 (20%)	28 (56%)	7 (14%)
**The course helps enhance my learning motivation**	**EBM-BOPPPS**	0 (0%)	2 (4.3%)	7 (14.9%)	28 (59.6%)	10 (21.3%)
	**BOPPPS**	0 (0%)	2 (4%)	17 (34%)	25 (50%)	6 (12%)
**The course develops my problem-solving skills**	**EBM-BOPPPS**	0 (0%)	2 (4.3%)	10 (21.2%)	23 (48.9%)	12 (25.5%)
	**BOPPPS**	1 (2%)	4 (8%)	19 (38%)	23 (46%)	3 (6%)
**The course promotes the memorization of knowledge**	**EBM-BOPPPS**	0 (0%)	1 (2.1%)	14 (29.8%)	25 (53.2%)	7 (14.9%)
	**BOPPPS**	0 (0%)	1(2%)	15 (30%)	20 (40%)	14 (28%)
**The course improves my communication skills**	**EBM-BOPPPS**	1 (2.1%)	2 (4.3%)	10 (21.3%)	28 (59.6%)	6 (12.8%)
	**BOPPPS**	1 (2%)	4 (8%)	9 (18%)	30 (60%)	6 (12%)
**The course improves my ability to give presentations**	**EBM-BOPPPS**	0 (0%)	2 (4.3%)	8 (17%)	29 (61.7%)	8 (17%)
	**BOPPPS**	0 (0%)	2 (4%)	6 (12%)	30 (60%)	12 (24%)
**I can formulate a clinical question to search the best evidence**	**EBM-BOPPPS**	0 (0%)	2 (4.3%)	12 (25.5%)	14 (29.8%)	19 (40.4%)
	**BOPPPS**	2 (4%)	4 (8%)	25 (50%)	10 (20%)	9 (18%)
**I am confident in critically appraising a journal article**	**EBM-BOPPPS**	0 (0%)	2 (4.3%)	10 (21.3%)	27 (57.4%)	8 (17%)
	**BOPPPS**	3 (6%)	4 (8%)	20 (40%)	17 (34%)	6 (12%)
**I consider evidence based medicine important to my future career**	**EBM-BOPPPS**	0 (0%)	1 (2.1%)	10 (21.3%)	15 (31.9%)	21 (44.7%)
	**BOPPPS**	1 (2%)	4 (8%)	21 (42%)	15 (30%)	9 (18%)
**I consider this course taking up too much of my preparation time**	**EBM-BOPPPS**	1 (2.1%)	15 (31.9%)	9 (19.1%)	15 (31.9%)	7 (14.9%)
	**BOPPPS**	7 (14%)	20 (40%)	10 (20%)	11 (22%)	2 (4%)
**I consider the preparation and presentation for this course is quite stressful for me**	**EBM-BOPPPS**	4 (8.5%)	11 (23.4%)	20 (42.6%)	4 (8.5%)	10 (21.3%)
	**BOPPPS**	10 (20%)	16 (32%)	14 (28%)	7 (14%)	3 (6%)

Note: Frequency is calculated as (number of responses in each category /total group size) × 100%
