# Supplementary material for: Effect of the EBM-integrated BOPPPS model on clinical competence and EBM confidence in neurology clerkships for three-year junior college medical clerks
Source: Front Public Health. 2025 Oct 13;13:1676073. doi: 10.3389/fpubh.2025.1676073 (PMC12554736; doi:10.3389/fpubh.2025.1676073)
Supplement: Supplementary file 2 [file Data_Sheet_2.PDF]

Students' Questionnaire

Dear students,

We are carrying out an evaluation to see if we can improve our teaching methods. Thank you for taking the time to fill in this questionnaire, it should only take 5 minutes. Your answers will be entirely anonymous. If you have any questions about this questionnaire, please feel free to contact Dr. Chen (cyx_4ever@163.com).

Best regards,
Chen Yaxi

....

Choose the number that most accurately describes your perception for each item.

	1 strongly disagree	2 disagree	3 neutral	4 agree	5 strongly agree
**It is easy to know the learning goals**	☐	☐	☐	☐	☐
**The course helps enhance my learning motivation**	☐	☐	☐	☐	☐
**The course develops my problem-solving skills**	☐	☐	☐	☐	☐
**The course promotes the memorization of knowledge**	☐	☐	☐	☐	☐
**The course improves my communication skills**	☐	☐	☐	☐	☐
**The course improves my ability to give presentations**	☐	☐	☐	☐	☐
**I can formulate a clinical question to search the best evidence**	☐	☐	☐	☐	☐
**I am confident in critically appraising a journal article**	☐	☐	☐	☐	☐
**I consider evidence based medicine important to my future career**	☐	☐	☐	☐	☐
**I consider this course taking up too much of my preparation time**	☐	☐	☐	☐	☐
**I consider the preparation and presentation for this course is quite stressful for me**	☐	☐	☐	☐	☐
**Anything else you want to say about this course?**					

Gender: male☐ female☐
