# Supplementary material for: Effect of the EBM-integrated BOPPPS model on clinical competence and EBM confidence in neurology clerkships for three-year junior college medical clerks
Source: Front Public Health. 2025 Oct 13;13:1676073. doi: 10.3389/fpubh.2025.1676073 (PMC12554736; doi:10.3389/fpubh.2025.1676073)
Supplement: Supplementary file 3 [file Data_Sheet_3.PDF]

Supplemental Teaching Manual

Teaching Manual for EBM-BOPPPS and BOPPPS Interventions

-Department of Neurology

1. General Information

Target population: 3-year junior college medical clerks (neurology rotation)

Duration: 2-month clerkship, 50-minute sessions (EBM-BOPPPS sessions on Wednesdays, BOPPPS sessions on Thursdays)

Instructors: 4 attending physicians (trained and certified via 2-hour workshop)

Core objective: Standardize delivery of EBM-BOPPPS and BOPPPS models

2. EBM-BOPPPS Intervention (6-Stage Protocol)

Stage	Timeline	Scripted Guidance for Instructors	Materials
**1. Bridge-in**	Day -3 (online)	"Please review the following ischemic stroke case (Case ID: IS-2024-01) and consider: What is the optimal antiplatelet therapy for this patient? Prepare 2-3 initial thoughts to share in groups."	- Clinical case: 65-year-old male with acute ischemic stroke (NIHSS=4), hypertension, no prior bleeding history<br>- Online discussion board link
**2. Objectives**	Day -3 (online)	"By the end of this session, you will be able to: (1) Formulate a PICO question for antiplatelet therapy in ischemic stroke; (2) Retrieve and appraise 2023 AHA/ASA guidelines; (3) Justify treatment decisions using evidence."	- Learning objective checklist (shared via online platform)

Stage	Timeline	Scripted Guidance for Instructors	Materials
**3. Pre-assessment**	5 mins (in-class)	"Let's start with 10 MCQs to check your baseline knowledge. Please submit answers via the classroom response system."	- MCQs: "Which is first-line antiplatelet for non-cardioembolic stroke? (A) Aspirin; (B) Clopidogrel; (C) Ticagrelor"<br>- Response system access code
**4. Participatory Learning**	35mins	"In groups of 4, use the library database to search for '2023 AHA/ASA ischemic stroke guidelines'. Focus on: (1) PICO elements in the case; (2) Recommendation strength for aspirin vs. dual antiplatelets; (3) Exclusion criteria. A representative will present your findings in 5 mins."	- Database access guide (PubMed, UpToDate)<br>- PICO worksheet (Patient: 65M with acute ischemic stroke; Intervention: Aspirin; Comparison: Dual antiplatelets; Outcome: Recurrent stroke at 90 days)
**5. Post-assessment**	5mins	"Complete the online quiz (5 questions) to test your understanding of guideline application. Example: 'When is dual antiplatelet therapy recommended in this case?'"	- Online quiz link (automatically scored, with instant feedback)
**6. Summary**	5mins	"Today we confirmed that aspirin is first-line for this patient (Class I, LOE A). Key takeaway: Always align treatment with patient-specific factors (e.g., bleeding risk) and latest guidelines. Please review the summary slide (shared online) for key references."	- Summary slide: Key guidelines, PICO formulation steps, and evidence hierarchy diagram

3. BOPPPS Control Intervention (6-Stage Protocol)

Stage	Timeline	Scripted Guidance for Instructors	Materials
**1. Bridge-in**	Day -3 (online)	"Please review the following ischemic stroke case (Case ID: IS-2024-01) and list possible antiplatelet therapies. Prepare to discuss in groups."	- Same clinical case as EBM-BOPPPS group
**2. Objectives**	Day -3 (online)	"By the end of this session, you will be able to: (1) Identify first-line antiplatelet therapies for ischemic stroke; (2) Describe dosing and contraindications."	- Learning objective checklist (no EBM focus)
**3. Pre-assessment**	5 mins (in-class)	"Let's start with 3 MCQs. Example: 'What is the standard dose of aspirin for stroke prevention?'"	- MCQs focused on drug facts (no guideline/appraisal content)
**4. Participatory Learning**	35 mins	"In groups of 4, discuss the case and propose a treatment plan. Focus on: (1) Drug selection; (2) Dosing; (3) Monitoring. A representative will present in 5 mins."	- No database access; reliance on textbooks/lecture notes
**5. Post-assessment**	5mins	"Complete the online quiz (5 questions) on antiplatelet dosing and side effects."	- Quiz focused on memorization (no evidence appraisal)
**6. Summary**	5mins	"First-line therapy for this case is aspirin 100mg/day. Key side effects: bleeding, gastritis. Review the drug fact sheet (shared online)."	- Drug fact sheet (no guidelines or evidence hierarchy)

4. Fidelity Monitoring Checklist

Item	Scoring (1=poor, 5=excellent)	Frequency of Assessment
Adherence to stage timelines	____	All sessions
Use of standardized case materials	____	Random 20% of sessions
Delivery of scripted guidance (key prompts)	____	Audio-recorded sessions
Alignment with learning objectives	____	Post-session instructor self-report

5.Standardized Case Information for 8 Shared Neurological Diseases

Disease Type	Case ID	Core Clinical Features (Consistent Across EBM-BOPPPS & BOPPPS Groups)	Key Focus for Interventions
Ischemic Stroke	IS-2024-01	65y male, acute onset right hemiplegia (NIHSS=4), hypertension, no bleeding history	EBM: 2023 AHA/ASA guideline application for antiplatelet therapy; BOPPPS: Antiplatelet dosing/contraindications
Hemorrhagic Stroke	HS-2024-02	58y female, sudden severe headache (VAS=9/10), CT-confirmed basal ganglia hemorrhage, history of untreated hypertension	EBM: Guideline-based blood pressure control targets; BOPPPS: Hemorrhage complication monitoring
Alzheimer's Disease	AD-2024-03	72y male, progressive memory decline	EBM: Evidence for cholinesterase inhibitor use;

Disease Type	Case ID	Core Clinical Features (Consistent Across EBM-BOPPPS & BOPPPS Groups)	Key Focus for Interventions
		(MMSE=22/30), functional impairment in daily activities	BOPPPS: Drug efficacy and adverse reactions
Viral Encephalitis	VE-2024-04	32y female, fever (39.2℃), headache, focal seizures, CSF showing lymphocytic pleocytosis	EBM: Antiviral therapy timing per IDSA guidelines; BOPPPS: CSF test interpretation and seizure management
Trigeminal Neuralgia	TN-2024-05	45y male, paroxysmal right facial pain (triggered by chewing), no neurological deficit	EBM: First-line pharmacotherapy (carbamazepine) evidence; BOPPPS: Drug dosage adjustment and side effects
Epilepsy	EP-2024-06	28y female, generalized tonic-clonic seizures (2 episodes/month), normal brain MRI	EBM: AED selection for newly diagnosed epilepsy; BOPPPS: AED adherence and drug interactions
Migraine	MG-2024-07	35y female, unilateral throbbing headache (VAS=8/10), associated with photophobia	EBM: Acute vs. preventive therapy evidence; BOPPPS: Migraine trigger identification and abortive drug use
Multiple Sclerosis	MS-2024-08	26y female, bilateral lower limb weakness, MRI showing periventricular T2 hyperintensities	EBM: Disease-modifying therapy (DMT) initiation criteria; BOPPPS: DMT administration and monitoring
